# Effect of aclidinium bromide therapy on quality of life and symptoms of patients with chronic obstructive pulmonary disease: pooled analysis results of the Greek and Austrian ON-AIR real-world observational studies

**DOI:** 10.1186/s12890-026-04183-5

**Published:** 2026-02-28

**Authors:** Horst Olschewski, Nikolaos Tzanakis, Stephan Brecht, Eva Wallner, Ioanna Kassianou, Panagiota Styliara, Linda Landini, Dalia Mostafa Kamal, Fabrizio Mazzicone, Konstantinos Kostikas

**Affiliations:** 1 Pneumology and Experimental Medicine, Sigmund Freud Private University, Freudplatz 1, Vienna, 1020 Austria; 2 Department of Pneumology, Intensive Care Medicine and Sleep Medicine, Charité University Medicine, Berlin, Germany; 3 Department of Thoracic Medicine, Medical School, University Hospital of Heraklion, University of Crete, Heraklion, Greece; 4 Menarini Pharma GmbH, Vienna, Austria; 5 Menarini Hellas, Athens, Greece; 6 Menarini Group, Florence, Italy; 7 Respiratory Medicine Department, University of Ioannina, Ioannina, Greece

**Keywords:** COPD assessment test, Daily activities, Predictors, LAMA, Night-time symptoms, Early-morning symptoms.

## Abstract

**Background:**

Chronic obstructive pulmonary disease (COPD) is a progressive respiratory condition characterized by persistent airflow limitation and debilitating symptoms that impair patients’ quality of life (QoL) and daily functioning. Aclidinium bromide is a long-acting muscarinic antagonist approved as a maintenance bronchodilator to relieve symptoms in adults with COPD. While randomized controlled trials have established the efficacy of aclidinium, real-world data are essential for understanding its effectiveness in routine clinical practice.

This pooled analysis of data from two prospective observational studies conducted in Austria and Greece aimed to supplement real-world evidence on the effects of aclidinium bromide on QoL, symptoms, and activity impairment of patients with COPD.

**Methods:**

Eligible participants were ≥40 years old and newly initiated on aclidinium bromide as monotherapy or add-on therapy, according to the approved product’s label. Patient-reported COPD assessment test (CAT), severity of night-time and early-morning symptoms, and interference of COPD with patients’ daily activities were assessed at enrollment and the 12-week follow-up visit.

**Results:**

One thousand one hundred twenty-seven patients (median age: 65.0 years, 65.8% male; 29.7% newly diagnosed) were enrolled between May 31st, 2013 and January 29th, 2016. Aclidinium bromide was initiated as monotherapy in 40.2% of patients. The median (interquartile range [IQR]) baseline CAT score decreased from 17.0 (11.0–23.0) to 11.0 (7.0–17.0) points (*p <* 0.001) after 12 weeks of treatment, with 82.7% of patients achieving a ‘minimal clinically important difference’ (MCID, i.e., ≥ 2-point decrease). The severity of night-time and early-morning symptoms significantly decreased over the 12-week follow-up period (*p <* 0.001 for both), while 51.9% of patients with ‘at least moderate impact’ of COPD on their daily activities at enrollment shifted to ‘small or no impact’ at the follow-up visit. Treatment with aclidinium was more likely to provide a clinically meaningful improvement in patients’ symptoms in patients who were treatment-naïve [adjusted odds ratio (OR): 2.14, 95% confidence interval (CI): 1.42–3.22; *p* < 0.001] and in those who were more symptomatic at baseline (OR: 1.13, 95% CI: 1.10–1.16; *p* < 0.001).

**Conclusions:**

This analysis of real-world data describes detailed aclidinium treatment benefit on COPD patients’ QoL, symptom severity and daily activity impairment.

**Trial registration:**

The Austrian ON-AIR trial was registered in a national Austrian NIS register with trial registration number NIS004104. The Greek ONAIR trial was registered on the SFEE’s (Hellenic Association of Pharmaceutical Companies) “dilon” registry, with trial registration number MeGR/14/BreCOPD/001.

**Supplementary Information:**

The online version contains supplementary material available at 10.1186/s12890-026-04183-5.

## Background

In 2021, the World Health Organization (WHO) ranked COPD as the fourth leading cause of mortality worldwide [1], and with the aging of the world population, in combination with continued exposure to risk factors, the burden of COPD is expected to increase over the coming decades [2].

COPD symptoms are associated with deteriorated health-related quality of life (HRQoL) and overall health status [3, 4], and can display seasonal and diurnal variability, with the early morning described as the worst and most troublesome time of day [5, 6], followed by night [7, 8]. The estimated proportion of COPD patients experiencing nocturnal symptoms and awakenings due to COPD is estimated to exceed 75% [9]. Overall, the symptom burden contributes to an increased risk of exacerbations and a worse prognosis among COPD patients [10–12]. In light of their importance in COPD, as well as their potential under-reporting by the patients, the 2025 Global initiative for chronic Obstructive Lung Disease (GOLD) suggests evaluation of symptoms, along with exacerbation history and comorbidities, separately from airflow limitation, and highlights their consideration when making therapeutic decisions [13].

COPD management mainly aims to prevent and control symptoms, reduce the frequency and severity of exacerbations, and preserve lung function, thereby improving patients’ functional status and HRQoL [13]. According to GOLD, individualized treatment regimens according to patients’ needs, with mainstay maintenance treatment consisting of dual bronchodilation with a long-acting muscarinic antagonist (LAMA) and a long-acting β2-agonist (LABA) represent the best treatment options [13]. However, there is also broad evidence for the beneficial effects of LAMA monotherapy in COPD [14–17].

Aclidinium bromide is a LAMA approved as a maintenance bronchodilator treatment in adults with COPD [18]. The drug is delivered through a multidose device-metered dry powder inhaler (Genuair^®^). The device has several features to ensure appropriate use and prevent accidental overdose [19]. Aclidinium is a potent inhibitor of all muscarinic receptor subtypes (M1-5), with a rapid onset and long-lasting bronchodilation action [20]. Its half-life for binding to the M3 receptor is approximately six times longer than its binding to the M2 receptor, which offers an advantage over other muscarinic receptor antagonists concerning the risk of M2-mediated cardiac side effects [19]. The safety profile of aclidinium bromide and its efficacy in improving lung function have been demonstrated in randomized controlled trials [15, 16, 21–24]. In addition, the beneficial effects of aclidinium bromide on patients’ HRQoL and COPD symptoms have been demonstrated in real-life settings across different countries [25–28].

The effects of aclidinium bromide on disease-specific HRQoL have not been extensively explored. The present work aimed to analyze the pooled data of two real-world observational studies carried out in Austria and Greece, focusing on the effect of aclidinium bromide on the health status, as assessed by the COPD assessment test (CAT), early-morning and night-time symptoms and interference of COPD with daily activities, also aiming to identify predictors of their improvement. Moreover, the evaluation of the aclidinium bromide Genuair^®^ inhaler by both patients and physicians was analyzed in the pooled sample.

## Methods

### Study design and setting

This is a pooled analysis of data collected in the context of two single-country, multi-center, non-interventional, prospective, observational studies of similar design conducted in Austria [27], and Greece [28]. The national competent ethics committees approved the study protocols, and the study was conducted following the International Society for Pharmacoepidemiology guidelines for Good Pharmacoepidemiology Practice [29], the ethical principles of the Declaration of Helsinki, and all standing regulations of each participating country. In the pooled cohort, the first patient was enrolled on May 31st, 2013 (Austrian study), while the last patient was enrolled on January 29th, 2016 (Greek study).

Patients were treated with aclidinium bromide for 12 weeks, according to the summary of product characteristics (SmPC). The physician’s decision to prescribe aclidinium bromide to a patient was not connected to their decision to include the patient in the study and preceded the patient’s enrollment. All aspects of patient management and monitoring followed standard medical practice. Study-related data were collected at enrollment and at the 12-week follow-up visit. Signed informed consent was obtained from all patients.

### Eligibility criteria and setting

The two studies included patients with an established COPD diagnosis, who were at least 40 years of age and newly initiated on treatment with aclidinium bromide inhalation powder, either as single or as add-on therapy [27, 28]. Main exclusion criteria included contraindications to the use of aclidinium bromide, pregnancy, and lactation.

Patients in Austria were recruited by 195 medical practices, including general practitioners, internists, and lung specialists nationwide. In contrast, patients in Greece were recruited from 15 hospital outpatient pulmonary clinics with a diverse geographic distribution across the country.

### Assessments

COPD’s impact on health status was assessed using the CAT [30]. The ‘minimal clinically important difference’ (MCID) in CAT corresponds to a 2-point decrease in total score [31, 32]. Patients were also asked to report the frequency of nocturnal sleep interruptions and to evaluate the severity of COPD night-time symptoms using a five-point Likert scale ranging from ‘no symptoms’ to ‘very severe symptoms’. A similar scale was used to evaluate the severity of early-morning symptoms and the burden of the most troublesome, namely cough, difficulty breathing, wheezing/breath sounds, and difficulty clearing mucus. The extent to which COPD symptoms impacted patients’ daily activities was also indicated on a five-point Likert scale ranging from ‘not at all’ to ‘a very large extent’. All the aforementioned outcomes were reported at enrollment and at the 12-week follow-up visit post-enrollment.

At the follow-up visit, patients and physicians indicated their willingness and intention to continue treatment with aclidinium bromide and evaluated the key features of the Genuair^®^ inhaler on a four-point Likert scale (very good, good, neutral, bad).

All adverse events experienced by the participating patients throughout the study observation period were adequately documented and reported.

### Statistical considerations

The normal distribution of continuous variables was examined using the Shapiro-Wilk test, with mean (standard deviation; SD) values presented when the data followed a normal distribution and median (interquartile range; IQR) when not. The statistical significance of differences of continuous variables was examined using the paired t-test or the Wilcoxon signed rank test, as applicable.

The impact of patient, disease, and treatment characteristics on achievement of MCID in the total CAT score and improvement of night-time symptoms, early-morning symptoms, and daily activities at the follow-up visit were evaluated using logistic regression models. Improvement in night-time and early-morning symptoms was defined as switching from having ‘at least moderate symptoms’ at enrollment to having ‘mild or no symptoms’ at the follow-up visit. Improvement in daily activities was defined as a shift to a category denoting less interference extent at the follow-up visit compared to enrollment. The multivariable logistic regression model was derived using a stepwise procedure based on the minimization of the Akaike’s Information Criterion and included the following baseline variables in the initial stepwise procedure: sex, age category, obesity [body mass index (BMI) ≥ 30 kg/m^2^], smoking status, newly-diagnosed COPD at the study visit, presence of cardiac/vascular and/or metabolism/nutrition disorders, comorbidity count, CAT total score at enrollment, no prior LAMA use, initiation of aclidinium bromide as add-on therapy with other inhaled maintenance therapy, and ‘at least moderate’ early-morning and/or night-time COPD symptoms. Aiming to control for potential differences between the two participating countries, the analysis above included ‘country’ as an independent covariate in the initial stepwise procedure. The correlation of CAT score at enrollment with CAT score at the 12-week visit was analyzed using Pearson’s correlation coefficient. The optimal cut-off value (for baseline CAT score) for achievement of MCID was estimated using receiver operating characteristic (ROC) analysis based on the maximization of the Youden index [33–35].

All statistical tests were two-sided and performed at a 0.05 significance level. Statistical analysis was conducted using SAS^®^ statistical analysis software (v.9.4; SAS Institute, Cary, NC).

## Results

### Patient population

Of the 843 patients recruited in the Austrian study, one patient was excluded from the analysis due to prior exposure to aclidinium bromide. Of the 286 patients enrolled in the Greek study, one patient was excluded due to a violation of the age eligibility criterion, while three patients from Austria aged < 40 years were included in the analysis. The resulting complete analysis set of the pooled population comprised 1127 patients, 842 (74.7%) were enrolled in Austria and 285 (25.3%) in Greece. The median (IQR) duration of follow-up in the overall analyzed population was 3.0 (2.1–3.1) months. Details on patient disposition have been provided previously [27, 28].

Patient characteristics at enrollment are presented in Table 1. The male-to-female ratio was approximately 2:1. Among patients with available data, the majority were male (65.8%), overweight or obese (66.5%), and ever smokers (84.6%).

**Table 1:** Patient and disease characteristics of the pooled study population at enrollment

Patient characteristics	
Sex (*N* = 1115), n (%)	
Male	734 (65.8)
Age (*N* = 1125), [median (IQR)], years	65.0 (57.0–73.0)
Age category (*N* = 1125), n (%)	
35–39 years	3 (0.3)
40–55 years	224 (19.9)
56–65 years	339 (30.1)
66–75 years	364 (32.4)
> 75 years	195 (17.3)
BMI (*N* = 1121), [median (IQR)], kg/m^2^	26.7 (24.0–30.0)
BMI category (*N* = 1121), n (%)	
Underweight (BMI < 18.5 kg/m^2^)	16 (1.4)
Normal (18.5 ≤ BMI < 25 kg/m^2^)	360 (32.1)
Overweight (25 ≤ BMI < 30 kg/m^2^)	465 (41.5)
Obese (BMI ≥ 30 kg/m^2^)	280 (25.0)
Smoking status (*N* = 1111), n (%)	
Current smoker	479 (43.1)
Former smoker	461 (41.5)
Never smoker	171 (15.4)
Time since smoking cessation (*N* = 450), [median (IQR)], years	8.0 (3.0–15.0)
Newly diagnosed with COPD (*N* = 1123), n (%)	334 (29.7)
COPD duration (*N* = 1054), [median (IQR)], years	2.4 (0.0–8.0)
At least one comorbidity (*N* = 1127), n (%)	811 (72.0)
Comorbidities in ≥ 10% of patients per System Organ Class (*N* = 1127), n (%)	
Cardiac/Vascular disorders	620 (55.0)
Metabolism/Nutrition disorders	386 (34.3)
Comorbidities in ≥ 10% of the patients per Preferred Term (*N* = 1127), n (%)	
Hypertension	425 (37.7)
Diabetes mellitus	208 (18.5)
Coronary artery disease	170 (15.1)
Dyslipidemia	159 (14.1)

*Abbreviations*: *BMI* Body mass index, *COPD* Chronic obstructive pulmonary disease, *IQR* Interquartile range

Comorbidities were reported for 72.0% of the overall study population, with the most prevalent being cardiovascular (55.0%) and metabolism/nutrition (34.3%) disorders. The median (IQR) number of comorbid conditions per patient was 1.0 (0.0–2.0), with 46.7% (526/1127) of the patients having two or more comorbidities. Comorbidities reported for ≥ 10% of the population are presented in Table 1.

### COPD history and treatment

The median COPD duration at enrollment was 2.4 years, with 29.7% of the patients being newly diagnosed. Prior to aclidinium bromide initiation, 29.0% (*N* = 327) of the overall study population had not received other treatment for COPD, whereas 9.2% (*N* = 104) had been exposed to one drug class, 25.0% (*N* = 282) to two drug classes, and 36.7% (*N* = 414) to three or more drug classes. The most frequently recorded drug classes of prior therapy were LABA (received by 50.0%), inhaled corticosteroids (ICS, received by 48.2%), and LAMA (received by 33.5%). Prior therapies are presented in Table 2.

**Table 2:** Prior treatment for COPD per drug class

Prior treatment	Study group		
Drug class, *n* (%)	Overall (*N* = 1127)	Austria (*N* = 842)	Greece (*N* = 285)
LABA	563 (50.0)	384 (45.6)	179 (62.8)
ICS	543 (48.2)	370 (43.9)	173 (60.7)
LAMA	377 (33.5)	279 (33.1)	98 (34.4)
SABA	344 (30.5)	324 (38.5)	20 (7.0)
SAMA	287 (25.5)	267 (31.7)	20 (7.0)
Xanthines	56 (5.0)	52 (6.2)	4 (1.4)
LTRA	17 (1.5)	13 (1.5)	4 (1.4)
Oral steroid	15 (1.3)	14 (1.7)	1 (0.4)
PDE4 inhibitor	9 (0.8)	5 (0.6)	4 (1.4)
Mucolytics	9 (0.8)	7 (0.8)	2 (0.7)
Antihistamines	3 (0.3)	1 (0.1)	2 (0.7)
Intranasal steroid	1 (0.1)	.	1 (0.4)
Not Specified	1 (0.1)	.	1 (0.4)
No prior COPD treatment	327 (29.0)	251 (29.8)	76 (26.7)

*Abbreviations*: *COPD* Chronic obstructive pulmonary disease, *ICS* Inhaled corticosteroids, *LABA* Long-acting β agonists, *LAMA* Long-acting muscarinic antagonists, *LTRA* Leukotriene receptor antagonist, *SABA* Short-acting β agonists, *SAMA* Short-acting muscarinic antagonists, *PDE4* Phosphodiesterase 4

Aclidinium bromide was initiated as monotherapy in 40.2% (440/1095) and as add-on therapy in 59.8% (655/1095) of patients; among the latter, inhaled maintenance therapies (LABA and/or LAMA and/or ICS) were co-administered in 80.2% (517/645) of the patients, and the most frequent combination was ICS and LABA, received by 69.5% (448/645) (as fixed-dose combination in most cases [66.5%; 429/645]).

### Improvement in health status assessed by CAT

**Fig. 1:**
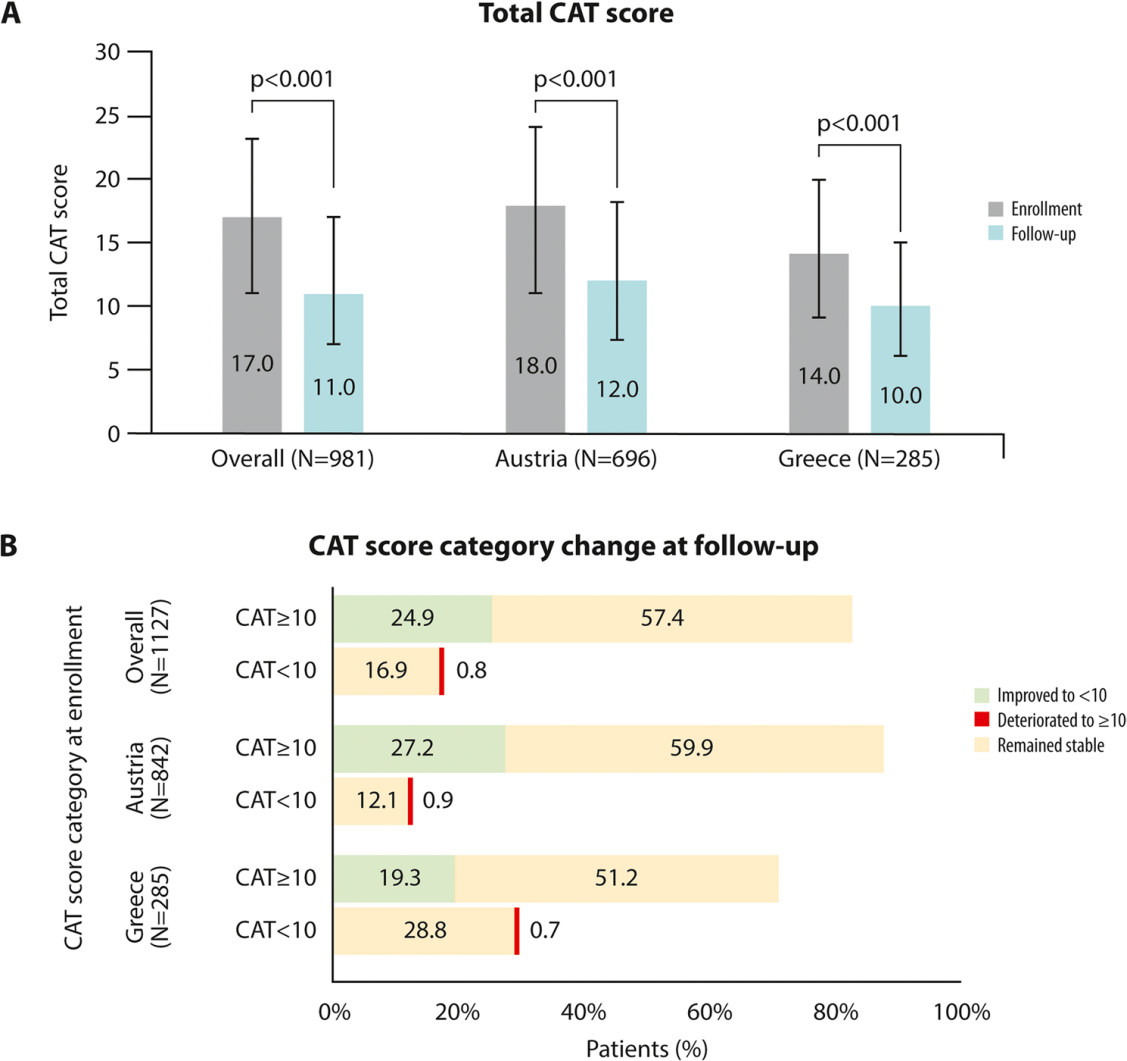
COPD assessment test (CAT) score at enrollment and at the 12-week follow-up visit. **A** Median (IQR) score is presented overall and per country; **B** Patient distribution in different categories according to their CAT scores at enrollment and at the follow-up visit. Green indicates an improvement from ≥ 10 at enrollment to < 10 at follow-up, red indicates a deterioration from < 10 at enrollment to ≥ 10 at follow-up, and yellow indicates that the score remained stable from enrollment to follow-up. The sum of the individual percentages adds up to 100%

A significant improvement in the CAT score was observed at follow-up after 12 weeks of treatment (Fig. 1A). At enrollment, the median (IQR) total CAT score was 17.0 (11.0–23.0) points among patients with available CAT data (*N* = 1007), with 81.9% (825/1007) of patients having a score ≥ 10 points. At the 12-week follow-up visit, CAT data was available for 984 patients, and the median (IQR) total CAT score was 11.0 (7.0–17.0), with 58.3% (574/984) of the patients having a score ≥ 10 points.

Overall, 82.7% (811/981) of the patients achieved at least a 2-point improvement in their CAT score, corresponding to the MCID. Of the patients with a CAT score ≥ 10 at enrollment, 30.2% (244/807) attained a score < 10 at the follow-up visit (Fig. 1B). Statistically significant (*p <* 0.001) reductions from enrollment were observed in all individual CAT item scores at the follow-up visit (Table 3).

**Table 3:** Change in CAT item scores between enrollment and 12-week visit in the overall population

CAT item	Mean (SD) reduction	Median (IQR) reduction	*p*-value of change
Cough frequency (*N* = 981)	0.8 (0.8)	1.0 (0.0–1.0)	< 0.001
Amount of phlegm (*N* = 981)	0.7 (0.9)	1.0 (0.0–1.0)	< 0.001
Chest tightness (*N* = 980)	0.6 (0.9)	0.0 (0.0–1.0)	< 0.001
Breathlessness walking uphill/upstairs (*N* = 980)	0.9 (0.9)	1.0 (0.0–1.0)	< 0.001
Limited in-home activities (*N* = 980)	0.6 (0.9)	0.0 (0.0–1.0)	< 0.001
Confident leaving home (*N* = 980)	0.3 (0.8)	0.0 (0.0–1.0)	< 0.001
Sleeping soundly (*N* = 980)	0.5 (0.9)	0.0 (0.0–1.0)	< 0.001
Lack of energy (*N* = 980)	0.6 (0.9)	1.0 (0.0–1.0)	< 0.001

*Abbreviations*: *CAT* COPD assessment test, *IQR* Interquartile range, *SD* Standard deviation

CAT score and reductions from enrollment at the follow-up visit were also examined in selected subgroups of interest (Supplementary Fig. 1).

### Improvement in night-time and early-morning COPD symptoms

**Fig. 2:**
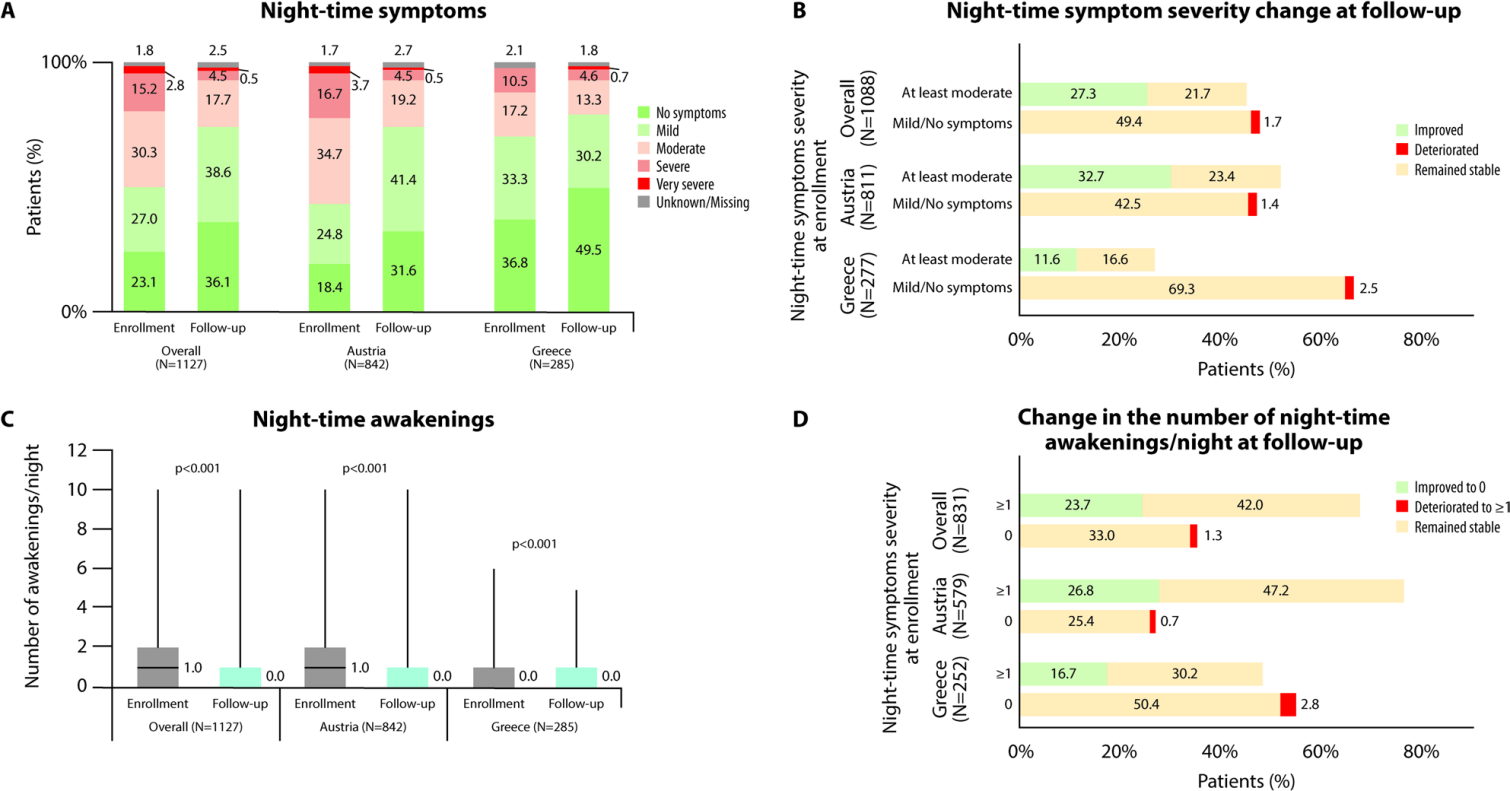
Severity of night-time symptoms at enrollment and at the 12-week follow-up visit. **A** Patient distribution according to the physician-assessed severity of night-time symptoms at enrollment and at the follow-up visit. Numbers indicate the percentages of patients; **B** Patient distribution in different categories according to their physician-assessed severity of night-time symptom category at enrollment and at the follow-up visit. Green indicates an improvement from ‘at least moderate severity’ at enrollment to ‘mild or no symptoms’ at follow-up, red indicates a deterioration from ‘mild or no symptoms’ at enrollment to ‘at least moderate severity’ at follow-up, and yellow indicates that the severity symptoms remained stable from enrollment to follow-up. The sum of the individual percentages adds up to 100%; **C** Median number of COPD-related awakenings per night, as reported by the patients. Boxes extend from 25th to 75th percentile, the line in the middle of the box represents the median, and bars extend from minimum to maximum in each category; **D** Patient distribution in different categories according to the number of night-time awakening/night at enrollment and at the follow-up visit. Green indicates an improvement from ≥ 1 awakenings at enrollment to no awakenings at follow-up, red indicates a deterioration from no awakenings at enrollment to ≥ 1 awakening at follow-up, and yellow indicates that the number of awakenings remained stable from enrollment to follow-up. The sum of the individual percentages adds up to 100%

The proportion of patients with night-time COPD symptoms decreased significantly from 76.5% (847/1107) at enrollment to 63.0% (692/1099) at the 12-week follow-up visit (*p <* 0.0001, Fig. 2A). COPD severity score, based on physicians’ assessment, was significantly reduced from enrollment to the follow-up visit (*p <* 0.001). Of the patients with ‘at least moderate’ night-time symptoms at enrollment, 55.7% (297/533) improved to having ‘mild or no symptoms’ at the follow-up visit. In comparison, only 3.2% (18/555) of those with ‘mild or no night-time symptoms’ at enrollment deteriorated to having ‘at least moderate symptoms’ at the follow-up visit (Fig. 2B). Among patients with available paired data, the frequency of nocturnal awakenings due to COPD decreased significantly (*p <* 0.001) from enrollment to the follow-up visit (Fig. 2C-D).

**Fig. 3:**
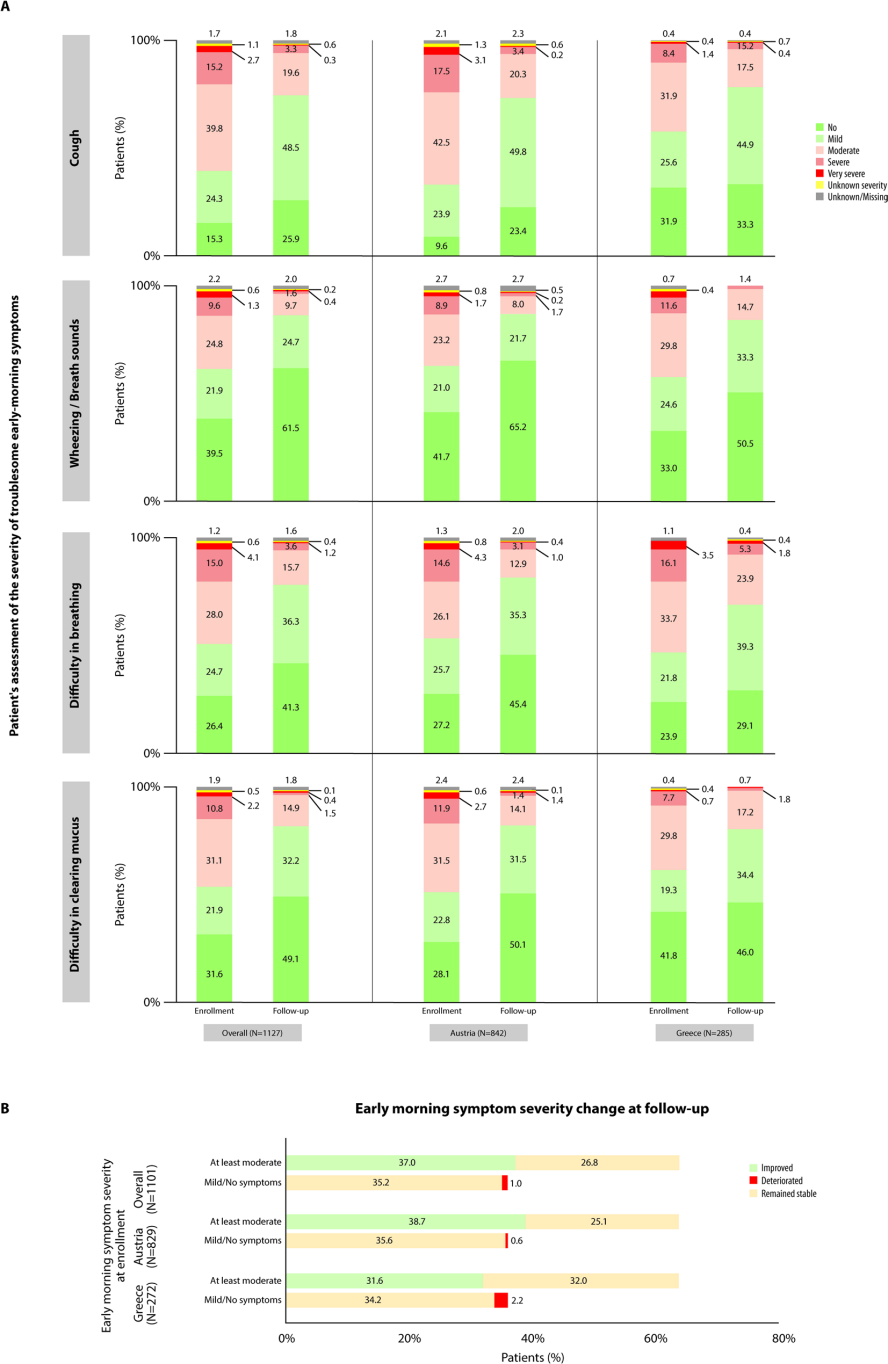
Severity of early-morning symptoms at enrollment and at the 12-week follow-up visit. **A** Patient distribution according to the severity of troublesome early-morning symptoms perceived by the patients at enrollment and at the follow-up visit. Numbers indicate the percentages of patients; **B** Patient distribution in different categories according to their physician-assessed overall early-morning symptom severity category at enrollment and at the follow-up visit. Green indicates an improvement from ‘at least moderate severity’ at enrollment to ‘mild or no symptoms’ at follow-up, red indicates a deterioration from ‘mild or no symptoms’ at enrollment to ‘at least moderate severity’ at follow-up, and yellow indicates that the severity of symptoms remained stable from enrollment to follow-up. The sum of the individual percentages adds up to 100%

Moreover, the proportion of patients with early-morning COPD symptoms decreased from 96.9% (1083/1118) at enrollment to 88.7% (979/1104) at the follow-up visit. The distribution of patients according to the self-reported severity of each early-morning symptom at enrollment and 12 weeks post-enrollment is presented in Fig. 3A. Among patients with paired assessments, based on physician assessment, the Likert severity scale improved significantly (*p <* 0.001) by median (IQR) 1.0 (0.0–1.0) point from enrollment to the follow-up visit and 58.0% (407/702) of those with ‘at least moderate’ early-morning symptoms at enrollment improved to ‘mild or no symptoms’ at the follow-up visit, while only 2.8% (11/399) of those with ‘no or mild symptoms’ at enrollment deteriorated to ‘at least moderate symptoms’ at the follow-up visit (Fig. 3B).

### Interference of COPD symptoms with patients’ daily activities

**Fig. 4:**
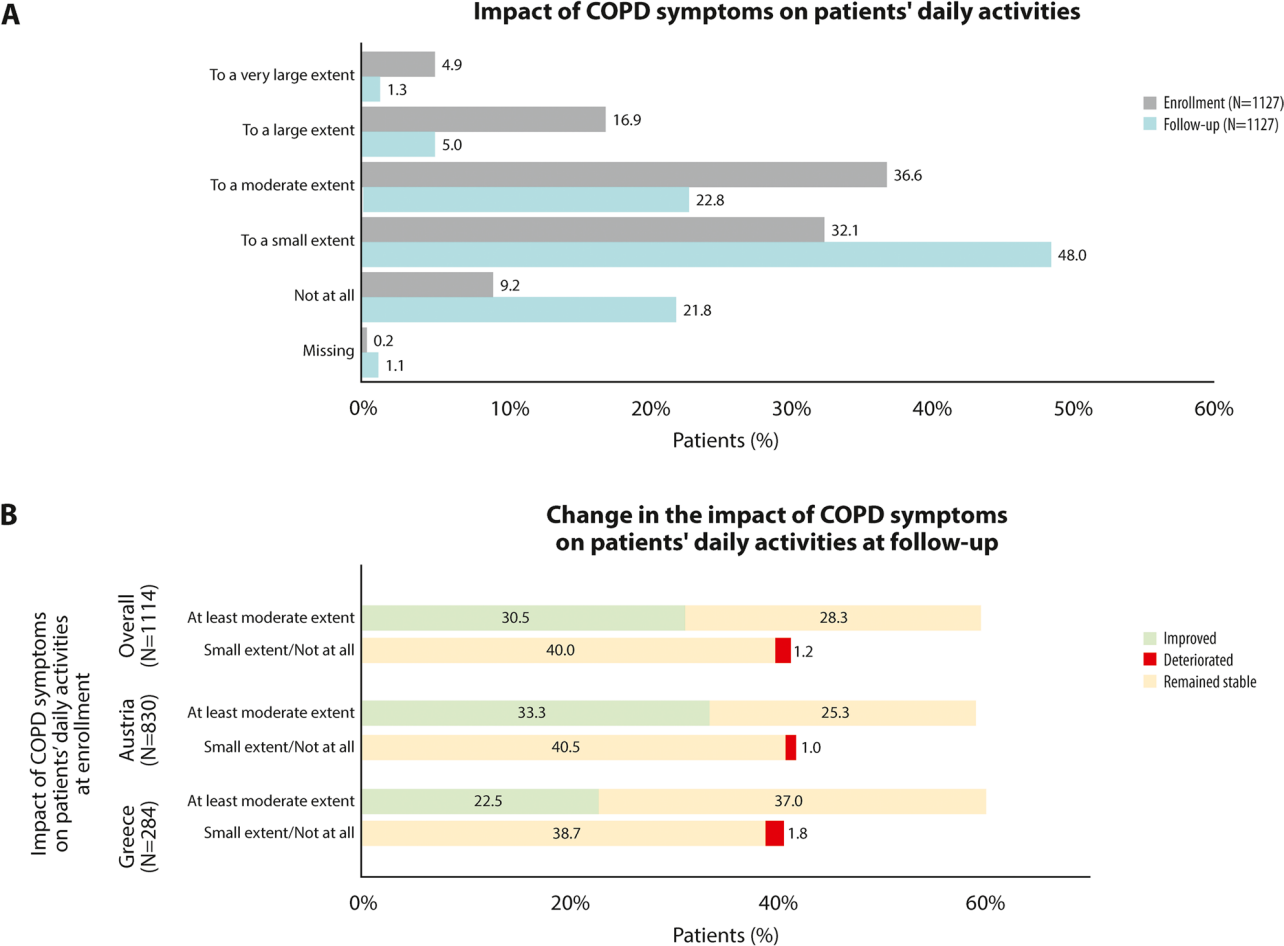
Impact of COPD symptoms on patients’ daily activities at enrollment and at the 12-week follow-up. **A** Patient distribution according to the impact of COPD symptoms on the patients’ daily activities at enrollment and at the 12-week follow-up visit; **B** Patient distribution in different categories according to the impact of their COPD symptoms on their daily activities at enrollment and at the 12-week follow-up visit. Green indicates an improvement from ‘at least moderate extent interference’ at enrollment to ‘small extent or no interference’ at follow-up, red indicates a deterioration from ‘small extent or no interference’ at enrollment to ‘at least moderate extent’ at follow-up, and yellow indicates that the interference remained stable from enrollment to follow-up. The sum of the individual percentages adds up to 100%

At enrollment, in the overall study population, 90.8% (1021/1125) of patients reported at least a small impact of COPD symptoms on their daily activities, while only 77.9% (869/1115) reported this at the follow-up visit (Fig. 4A). From enrollment to follow-up, 51.9% (340/655) of those with ‘at least moderate’ impact of COPD on their daily activities, improved to ‘small or no impact’, while only 2.8% (13/459) deteriorated (Fig. 4B).

### Evaluation of the aclidinium bromide Genuair^®^ inhaler by patients and physicians

**Fig. 5:**
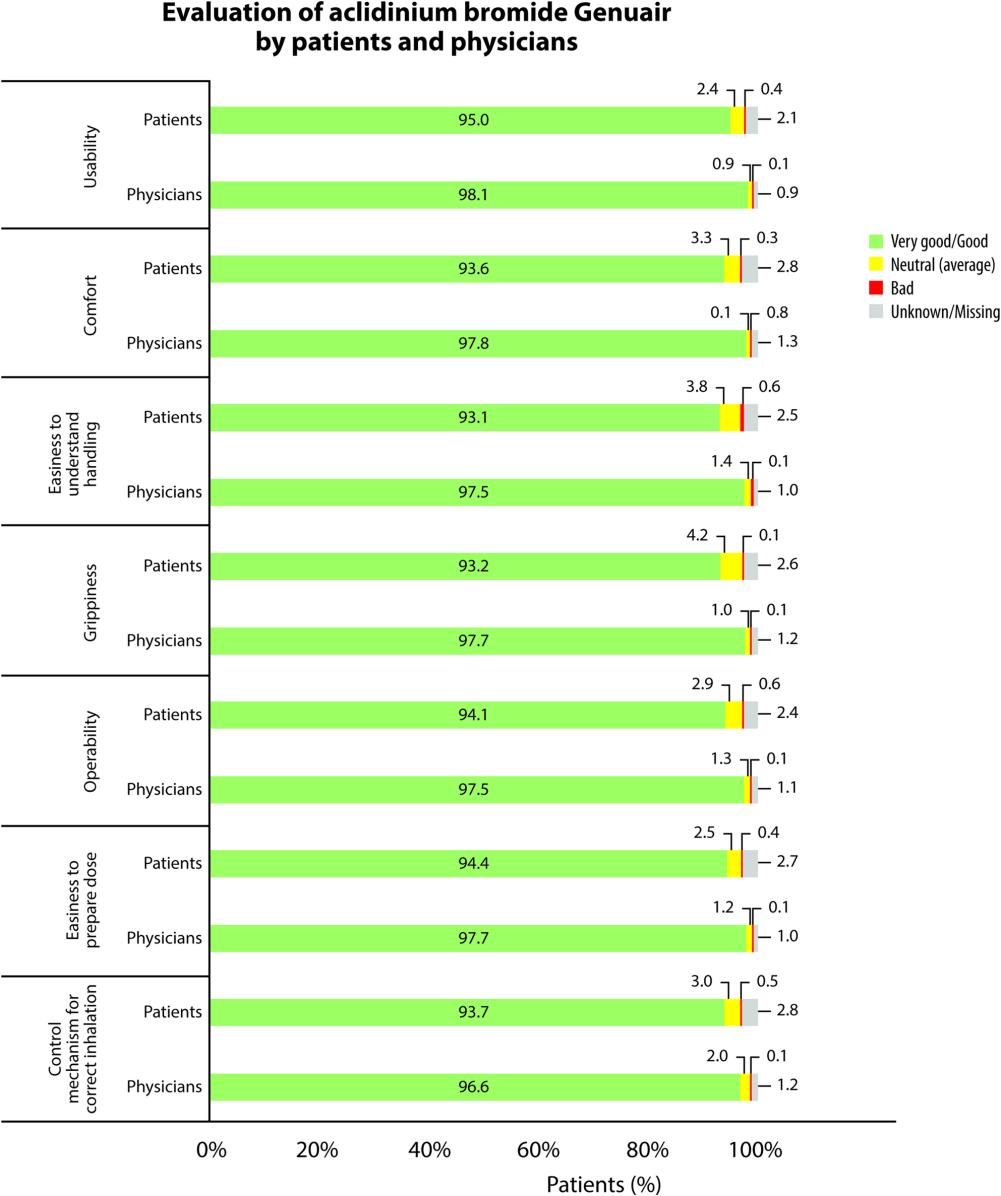
Evaluation of aclidinium bromide Genuair^®^ by patients and physicians. Proportions of patients/physicians ranking each of the examined features as very good/good, neutral, or bad

The ratings of the features of aclidinium bromide Genuair^®^ inhaler by patients and physicians are presented in Fig. 5. All features of Genuair^®^ were ranked as good or very good both by patients and physicians in more than 93.0% of the cases. Moreover, physicians reported that they intended to continue therapy with aclidinium bromide after the end of the study in 94.9% (1059/1116) of their patients. Similarly, 93.6% (1045/1117) of the patients reported their willingness to continue therapy after the end of the study. The top reasons for treatment continuation for both physicians and patients were good effectiveness, compatibility, and manageability.

### Predictors of improvement in COPD symptoms with aclidinium bromide treatment

The association of selected patient, disease, and treatment characteristics of interest with improvement in COPD symptoms at the 12-week follow-up visit was examined by univariate and multivariate logistic regression analysis. In particular, factors of interest, described in the ‘Materials and Methods’ section, were examined in terms of their association with the achievement of at least 2 points improvement (MCID) in total CAT score, as well as with the improvement in night-time and early-morning symptoms, and the effect of COPD symptoms on patients’ daily activities.

**Fig. 6:**
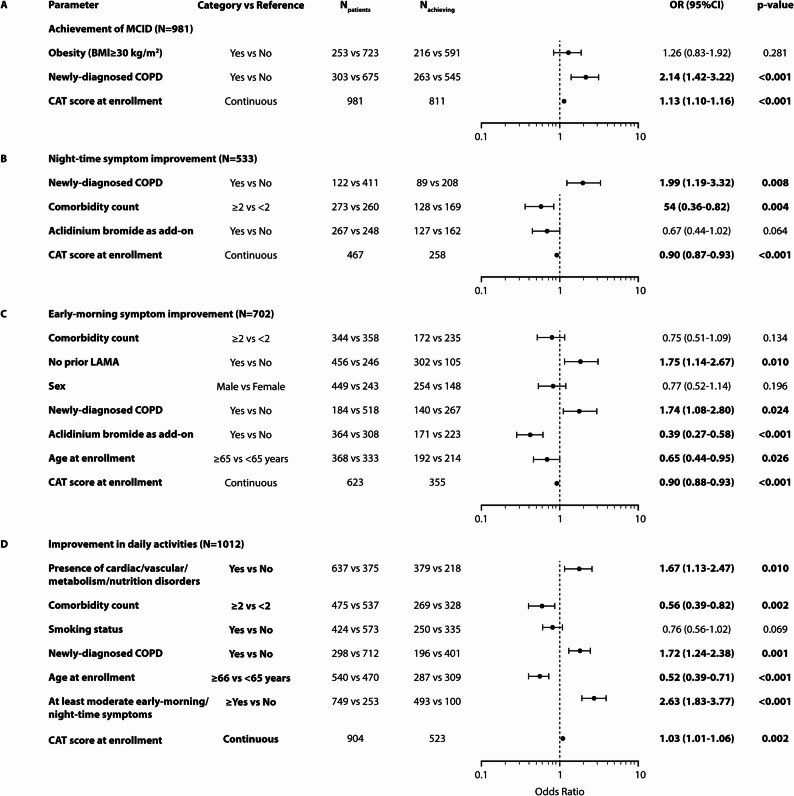
Multivariable regression analysis. Association of patient and disease characteristics with (**A**) Achievement of the ‘minimal clinically important difference’ (MCID) in the total COPD assessment test (CAT) score; **B** Improvement in night-time symptoms (*N* = 533 indicates the number of patients with at least moderate night-time symptoms at enrollment); **C** Improvement in early-morning symptoms (*N* = 702 indicates the number of patients with at least moderate early-morning symptoms at enrollment); and **D** Improvement in daily activities (*N* = 1012 indicates the number of patients with COPD symptoms that interfere with their daily activities to ‘at least a small extent’), from enrollment to the 12-week follow-up visit. CI, Confidence interval; OR, Odds ratio

Among the factors retained in the final multivariable model, newly-diagnosed COPD and CAT score at enrollment were significantly associated with MCID achievement at the follow-up visit (Fig. 6A). In particular, newly-diagnosed patients had approximately 2-fold higher odds of achieving MCID versus previously diagnosed patients [adjusted odds ratio (OR): 2.14; *p* < 0.001]. Moreover, the odds of MCID achievement at the follow-up visit increased by 13% for each one-point increase in total CAT score at enrollment (OR: 1.13; *p* < 0.001).

In addition, Pearson correlation analysis revealed a statistically significant strong, positive correlation of CAT score at enrollment with the respective score at the follow-up visit (rho = 0.84; *p <* 0.001). The optimal threshold of baseline CAT score for predicting MCID achievement, as calculated using the Youden Index, was 13 (sensitivity = 76%; specificity = 59%; Youden’s index = 0.351; Area Under the Curve = 0.710). Univariable logistic regression analysis demonstrated that patients with CAT score ≥ 13 at enrollment had more than 4-fold greater odds of achieving MCID improvement at 12 weeks compared to those with CAT score < 13 at enrollment (OR: 4.56, 95% CI: 3.23–6.45; *p* < 0.001).

Concerning night-time symptoms, newly-diagnosed COPD patients had approximately 2-fold higher odds of improving from ‘at least moderate symptoms’ at enrollment to ‘mild or no symptoms’ at the follow-up visit (OR: 1.99; *p* = 0.008) versus previously diagnosed patients. Interestingly, the odds of night-time symptom improvement at the follow-up visit decreased by 10% (OR: 0.90; *p* < 0.001) for every one-point increase in total CAT score at enrollment, and were 46% lower for patients with two or more comorbidities at enrollment (OR: 0.54; *p* = 0.004) versus patients with one or no comorbidity (Fig. 6B).

The analysis of the association of parameters of interest with improvement in early-morning symptoms revealed that patients newly diagnosed with COPD (OR: 1.74; *p* = 0.024) and patients who had not received prior LAMA treatment (OR: 1.75; *p* = 0.010) had higher odds of shifting from ‘at least moderate’ early-morning symptoms at enrollment to ‘mild or no symptoms’ at follow-up versus the rest, while patients who initiated aclidinium bromide as add-on with other inhaled maintenance treatment (OR: 0.39; *p* < 0.001), and those aged ≥ 65 years at enrollment (OR: 0.65; *p* = 0.026) had lower odds of achieving improvement in early-morning symptoms at follow-up (Fig. 6C). Moreover, for every one-point increase in CAT score at enrollment, the odds of early-morning symptom improvement at follow-up decreased by 10% (OR: 0.90; *p* < 0.001).

Selected factors of interest are associated with how COPD symptoms impact patients’ daily activities. Patients had significantly higher odds of shifting to a category denoting a lower impact after 12 weeks of treatment if they had cardiac/vascular and/or metabolism/nutrition disorders at enrollment (OR: 1.67; *p* = 0.010), if they were newly diagnosed with COPD (OR: 1.72; *p* = 0.001), or if they had ‘at least moderate’ early-morning and/or night-time COPD symptoms at enrollment (OR: 2.63; *p* < 0.001) (Fig. 6D). In addition, for every one-point increase in total CAT score at enrollment, the odds of alleviating the impact of COPD symptoms on daily activities increased by 3% (OR: 1.03; *p* = 0.002). In contrast, patients with two or more comorbidities at enrollment (OR: 0.56; *p* = 0.002) as well as patients aged ≥ 65 years at enrollment (OR: 0.52; *p* < 0.001) had significantly diminished odds of achieving a lower impact of COPD on their daily activities at follow-up (Fig. 6D).

Statistical significance and direction of the associations above were retained when analyzing the models including ‘country’ as a covariate (Supplementary Fig. 2). 

### Safety

Based on the results of the pooled safety analysis, none of the patients died and there were no life-threatening events, while 1.0% (11/1127) of the patients experienced a total of 19 adverse events during their observation in the study, 18 of which [experienced by 0.9% (10/1127) of the patients] were assessed to be causally related to aclidinium bromide, including one serious event of dry mouth (Table 4). The study drug-related events reported at the highest frequency included dry mouth, vomiting, and drug ineffectiveness, recorded for two patients each (0.2%). 

**Table 4:** Adverse events experienced in the overall population

Adverse event	*N* of events	*N* of patients (%)
Overall	19	11 (1.0)
Infections	1	1 (0.1)
Laryngitis	1	1 (0.1)
Eye disorders	1	1 (0.1)
Blurred vision	1	1 (0.1)
Ear and labyrinth disorders	1	1 (0.1)
Vertigo	1	1 (0.1)
Cardiac disorders	2	2 (0.2)
Tachycardia	1	1 (0.1)
Tachycardia paroxysmal	1	1 (0.1)
Respiratory, thoracic, and mediastinal disorders	6	4 (0.4)
Cough	1	1 (0.1)
Dysphonia	1	1 (0.1)
Dyspnea	1	1 (0.1)
Oropharyngeal pain	1	1 (0.1)
Pleural effusion	1 (serious)	1 (0.1)
Productive cough	1	1 (0.1)
Gastrointestinal disorders	5	5 (0.4)
Dry mouth	2 (1 serious)	2 (0.2)
Vomiting	2	2 (0.2)
Nausea	1	1 (0.1)
Skin and subcutaneous tissue disorders	1	1 (0.1)
Rash	1	1 (0.1)
General disorders and administration site conditions	2	2 (0.2)
Drug ineffective	2	2 (0.2)

## Discussion

The findings of this study show that 12-week treatment with aclidinium bromide in COPD patients significantly improves their health status according to their CAT score, night and morning COPD symptom severity, and how COPD symptoms interfere with daily activities, considerably improving their HRQoL. The most important predictors of achieving an MCID were having a higher CAT score at enrollment and being a newly-diagnosed patient.

The present analysis provides real-world evidence in a pooled sample of 1127 COPD patients treated in Austria and Greece’s routine care settings. While the pooled dataset requires physician-diagnosed COPD in aclidinium naïve out-patients only, it aims to include patients with various clinical presentations. There were distinct differences in the datasets from Austria and Greece, such as disease duration, status of current smokers, or prior ICS and LABA use, therefore ‘country’ was used as a control variable to establish the regression model [27, 28].

An initial CAT score of 17 at the beginning of the observation period revealed a symptom burden similar to controlled clinical trials not focusing on exacerbation reduction, such as SUMMIT [36], or other real-world studies [25, 37]. The aclidinium maintenance treatment within the different co-medications resulted in a statistically significant median reduction of 4.0 (mean: 5.1) points at the 12-week follow-up visit, consistent with the 4.7-point reduction reported by Marth et al. [25]. Importantly, the majority of patients (eight out of ten) in the pooled population achieved at least a symptom reduction equal or exceeding the MCID, while approximately one-third of those who had a CAT score ≥ 10 at enrollment, a cut-off generally used to categorize COPD as symptomatic and consider regular treatment [13]. attained a score < 10 at 12 weeks. Moreover, the observed reduction was statistically significant not only in the total CAT score but also for all eight single CAT items, indicating a widespread benefit of aclidinium across a broad range of effects of COPD on patients’ health, which is considered of great importance, particularly since individual items or subgroups of items may bring valuable insights beyond the total score and carry additional information regarding COPD characteristics [38].

Significant improvements following the twice daily aclidinium initiation were also observed regarding night-time and early-morning symptoms, as well as interference of COPD symptoms with patients’ daily activities, consistently with other real-world studies [25, 26], and randomized controlled trials [16, 17], and in line with an increase in exercise capacity following aclidinium therapy [39]. Indicatively, more than half of the patients with ‘at least moderate’ night-time symptoms at enrollment improved to ‘mild or no symptoms’ at follow-up, based on physicians’ assessment, and the number of nocturnal awakenings decreased significantly. Similarly, more than half of the patients reporting ‘at least moderate interference’ of COPD with their daily activities at enrollment reported ‘small or no impact’ of COPD at the follow-up visit. Concerning early-morning symptoms, a statistically significant reduction on the Likert scale, was noted between enrollment and follow-up, with approximately four out of ten patients with ‘at least moderate’ early-morning symptoms shifting to ‘mild or no symptoms’ over the 12 weeks post-enrollment. The general alignment between physicians’ and patients’ assessments strengthens the validity of the findings, as discrepancies in the perception of the two parties regarding global health status have been previously reported [40].

A strength of this study is the large number of patients, which was achieved by pooling two studies performed in different European countries. Given the heterogeneity in symptom severity and presentation among COPD patients, and in the context of the individualized treatment approach increasingly adopted by physicians, identification of patient, disease, and treatment characteristics that could serve as predictors of response to specific therapies is of utmost importance. The present analysis identified CAT score at enrollment and newly-diagnosed COPD as statistically significantly associated with higher odds of achieving MCID and improvement in daily activities at 12 weeks post-enrollment, a result consistent with previous studies demonstrating the beneficial effect of aclidinium in symptomatic patients [17]. The presence of cardiac/vascular and/or metabolic/nutritional disorders was associated with higher odds of experiencing an improvement in daily activities. Moreover, initiation of aclidinium as monotherapy maintenance treatment rather than as add-on therapy was associated with higher odds of experiencing an improvement in early-morning and night-time symptoms, even though the latter did not reach statistical significance. Previous data obtained in the randomized controlled trial setting indicated no statistically significant difference in the change in night-time and early-morning symptom severity between subgroups of patients treated with aclidinium bromide combined with the LABA formoterol fumarate and those treated with aclidinium bromide monotherapy [41]. Moreover, newly-diagnosed COPD was statistically significantly associated with higher odds of improvement in night-time and early morning symptoms, as well as daily activities. The above findings suggest that, at least for specific patient categories, e.g., newly-diagnosed and symptomatic patients, administration of aclidinium monotherapy may be a suitable initial therapeutic option, while treatment escalation strategies are more effective if the symptom burden is moderate to severe as compared to mild. In a broader context, early diagnosis and intervention in COPD is regarded by some experts as a largely unmet challenge that would be very critical to overcome, with the failure of several treatments being at least partly attributed to their administration late during the disease when lung damage is already extensive and irreversible [42].

Statistical significance of the association of the aforementioned parameters with QoL and symptom improvement was retained even when including ‘country’ as a potential confounder, which supports the generalizability of the observed findings across different healthcare settings and socioeconomic environments, and despite numerical differences between the two cohorts in the frequency of specific drug classes received as prior treatment.

There is also evidence from controlled studies (EMAX) that inhalation therapy with dual bronchodilators adds lung function benefits and results in clinical improvement in COPD patients compared to bronchodilator monotherapy [43]. Still, real-world evidence will help physicians define the place in therapy for LAMA therapy with aclidinium.

Limitations of the two studies analyzed herein have been described previously [27, 28], and are mainly attributed to their observational design and the lack of a comparator arm.

## Conclusions

The pooled analysis results provide further real-world evidence for the clinically meaningful and statistically significant effect of aclidinium treatment on quality of life, night-time, and early-morning symptom burden and activity-limitations associated with COPD. Treatment with aclidinium was more likely to provide a clinically meaningful improvement in patients’ symptoms in patients who were treatment-naïve and those who were more symptomatic at baseline. This analysis also confirms the drug’s favorable safety profile and the patients’ satisfaction with treatment in a large cohort from two European countries.

## Supplementary Material

Supplementary Material 1.

Supplementary Material 2

## Data Availability

No datasets were generated or analysed during the current study.

## Abbreviations

BMI: Body mass index
CAT: COPD assessment test
COPD: Chronic obstructive pulmonary disease
GOLD: Global initiative for chronic obstructive lung disease
HRQoL: Health-related quality of life
ICS: Inhaled corticosteroids
IQR: Interquartile range
LABA: Long-acting β2-agonist
LAMA: Long-acting muscarinic antagonist
LTRA: Leukotriene receptor antagonist
MCID: Minimal clinically important difference
OR: Adjusted odds ratio
PDE4: Phosphodiesterase 4
QoL: Quality of life
ROC: Receiver operating characteristic
SABA: Short-acting β agonists
SAMA: Short-acting muscarinic antagonists
SmPC: Summary of product characteristics
SD: Standard deviation
WHO: World health organization
