# Supplementary figures and images for: Effect of aclidinium bromide therapy on quality of life and symptoms of patients with chronic obstructive pulmonary disease: pooled analysis results of the Greek and Austrian ON-AIR real-world observational studies
Source: BMC Pulm Med. 2026 Feb 28;26:157. doi: 10.1186/s12890-026-04183-5 (PMC13059566; doi:10.1186/s12890-026-04183-5)

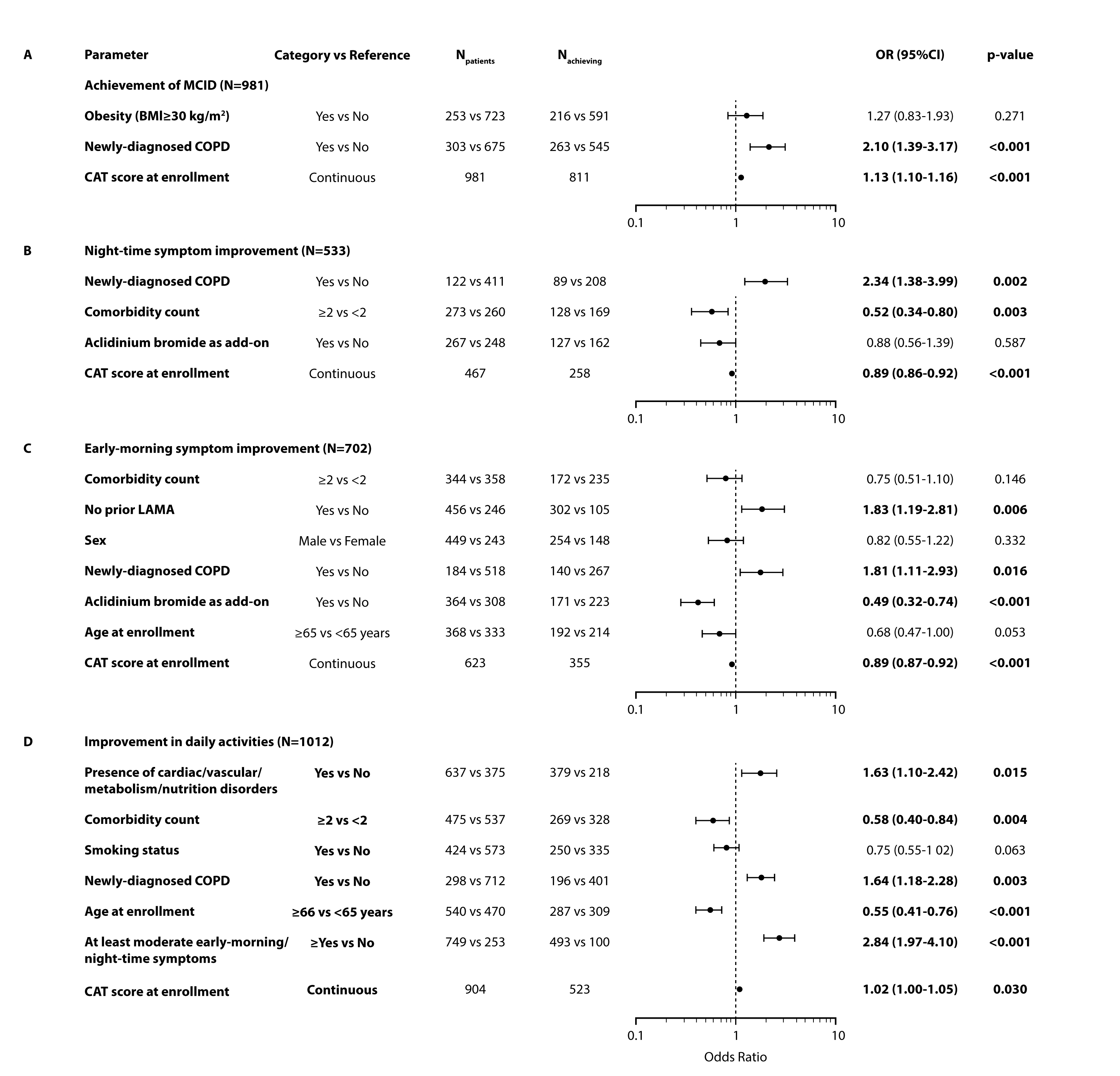

Supplement: Supplementary file 1 — Supplementary Material 1. [file 12890_2026_4183_MOESM1_ESM.tif]

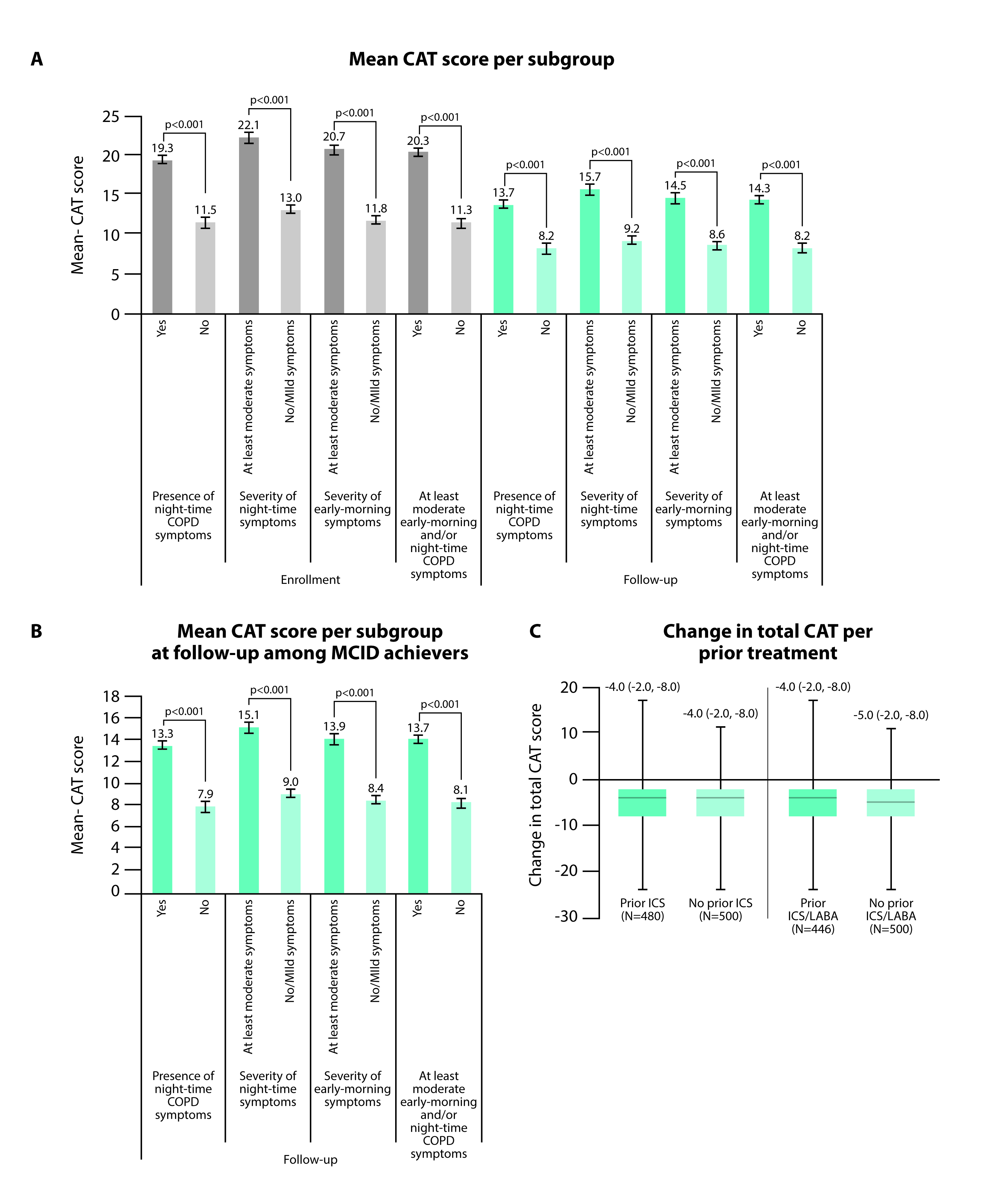

Supplement: Supplementary file 2 — Supplementary Material 2 [file 12890_2026_4183_MOESM2_ESM.tif]
